# Desiccation Induced Conjunctival Monocyte Recruitment and Activation - Implications for Keratoconjunctivitis

**DOI:** 10.3389/fimmu.2021.701415

**Published:** 2021-07-08

**Authors:** Jehan Alam, Cintia S. de Paiva, Stephen C. Pflugfelder

**Affiliations:** Ocular Surface Center, Department of Ophthalmology, Baylor College of Medicine, Houston, TX, United States

**Keywords:** monocyte, macrophage, conjunctiva, dry eye, keratoconjunctivitis sicca, Sjogren syndrome

## Abstract

**Background:**

Lacrimal gland secretory dysfunction in Sjögren syndrome (SS) causes ocular surface desiccation that is associated with increased cytokine expression and number of antigen-presenting cells (APCs) in the conjunctiva. This study evaluated the hypothesis that desiccating stress (DS) alters the percentage and gene expression of myeloid cell populations in the conjunctiva.

**Methods:**

DS was induced by pharmacologic suppression of tear secretion and exposure to drafty low humidity environment. Bone marrow chimeras and adoptive transfer of CD45.1^+^ bone marrow cells to CD45.2^+^ C-C chemokine receptor 2 knockout (CCR2^-/-^) mice were used to track DS-induced myeloid cell recruitment to the conjunctiva. Flow cytometry evaluated myeloid cell populations in conjunctivae obtained from non-stressed eyes and those exposed to DS for 5 days. CD11b^+^ myeloid lineage cells were gated on monocyte (Ly6C), macrophage (CD64, MHCII) and DC (CD11c, MHCII) lineage markers. NanoString immune arrays were performed on sorted MHCII^+^ and MHCII^-^ monocyte/macrophage cell populations.

**Results:**

DS significantly increased the recruitment of adoptively transferred MHCII positive and negative myeloid cells to the conjunctiva in a CCR2 dependent fashion. The percentage of resident conjunctival monocytes (Ly6C^+^CD64^-^) significantly decreased while CD64^+^MHCII^+^ macrophages increased over 5 days of DS (P<0.05 for both). Comparison of gene expression between the MHCII^-^ monocyte and MHCII^+^ populations in non-stressed conjunctiva revealed a ≥ 2 log^2^ fold increase in 95 genes and decrease in 46 genes. Upregulated genes are associated with antigen presentation, cytokine/chemokine, M1 macrophage and NLRP3 inflammasome pathways. DS increased innate inflammatory genes in monocytes and MHCII^+^ cells and increased M1 macrophage (Trem1, Ido1, Il12b, Stat5b) and decreased homeostasis (Mertk) and M2 macrophage (Arg1) genes in MHCII^+^ cells.

**Conclusions:**

There are myeloid cell populations in the conjunctiva with distinct phenotype and gene expression patterns. DS recruits myeloid cells from the blood and significantly changes their phenotype in the conjunctiva. DS also alters expression of an array of innate inflammatory genes. Immature monocytes in the unstressed conjunctiva appear to cascade to MHCII^+^ macrophages during DS, suggesting that DS promotes maturation of monocytes to antigen presenting cells with increased expression of inflammatory genes that may contribute to the pathogenesis of SS keratoconjunctivitis sicca.

## Introduction

Reduced tear secretion and loss of the ability to reflex tear in Sjögren syndrome (SS) results in significant reduction in tear volume, increased tear osmolarity and development of ocular surface disease termed keratoconjunctivitis sicca (KCS) (1–3). These changes activate stress signaling pathways in the ocular surface epithelial and immune cells that results in production of innate inflammatory mediators, including the monocyte chemokine, CCL2 (4). Recruitment of myeloid cells from the blood into the wounded intestinal mucosa and cornea of mice has been found to be CCR2 dependent (5–7). An increased percentage of CD11b^+^ myeloid cells in the conjunctiva and cornea has been reported in the desiccating stress-induced murine dry eye model that develops SS like ocular surface disease, but it has not been established if these cells are recruited from the blood in a CCR2 dependent manner (8, 9). An increase in antigen-presenting cells (APCs) and T cells in the conjunctiva of patients with SS KCS has been reported (10, 11). MHCII^+^ CD11b^+^ and CD11c^+^ cells were noted to increase in the ocular surface draining lymph nodes of mice exposed to experimental desiccating stress (DS) (12). These cells were found to prime autoreactive CD4+ T cells that cause KCS when adoptively transferred to naïve immunodeficient recipients (13–15). Myeloid cells produce the cytokine IL-12 that stimulates natural killer (NK) and CD4+ T cells to produce IFN-*γ*, a cytokine that causes conjunctival goblet cell loss in KCS (16–18). Increased IFN-*γ* expression and percentage of HLA-DR^+^ cells in the conjunctiva have been found to correlate with goblet cell loss and severity of KCS in patients with SS (11, 19). The myeloid cells in the conjunctiva have not been well characterized, but may consist of monocytes and macrophages that become activated by DS and produce mediators that participate in the pathogenesis of KCS. The purpose of this study is to characterize changes in phenotype and gene expression in conjunctival myeloid cell populations in response to DS.

## Methods

Animals and DS model. The animal protocol for this study was designed according to the ARVO Statement for the use of Animals in Ophthalmic and Vision Research and was approved by the Institutional Animal Care and Use Committee at Baylor College of Medicine.

### Mice

Female C57BL/6J (B6) (n=60), CCR2^-/-^ (n=30) and B6.SJL-Ptprc Pepc/BoyJ CD45.1^+^ (n=12) mouse strains aged 6–8 weeks were purchased from Jackson Laboratories (Bar Harbor, ME), and allowed to rest in a humidified environment for one week before the experiment. Pepc/BoyJ CD45.1^+^ (6–8-week-old) mice were used as adoptive transfer donors.

### Desiccating Stress

DS was induced by inhibiting tear secretion with scopolamine hydrobromide (Greenpark, Houston) in drinking water (0.5mg/mL) and housing in a cage with a perforated plastic screen on one side to allow airflow from a fan placed 6 inches in front of it for 16 h/day for 5 consecutive days. Room humidity was maintained at 20–30%. Control mice were maintained in a non-stressed (NS) environment at 50–75% relative humidity without exposure to an air draft.

### Adoptive Transfer and Creation of Bone Marrow Chimeras

Bone marrow cells were harvested from the femur and tibial bones of the Pepc/BoyJ strain and approximately 2 million cells in 0.1mL were injected into the right orbit of CD45.2^+^CCR2^-^/^-^ mice 3 times at 2-day intervals. Prior to the 2^nd^ injection, they were either maintained at ambient humidity or subjected to DS for 5 days. On day 5, mice were euthanized, the bulbar conjunctiva was excised, cell suspensions were prepared, incubated with anti-CD45.1^+^ (A20, Catalog no. 110717, Biolegend, San Diego, CA) and analyzed by flow cytometry. CD45.2^+^ CCR2 positive and negative bone marrow chimeras were created in the Pepc/BoyJ strain as previously reported (20). Ten days after bone marrow reconstitution, mice were subjected to DS and myeloid cells in the conjunctiva were analyzed by flow cytometry.

### Flow Cytometry

Conjunctivae were excised, chopped with scissors into tiny pieces and incubated with 0.1% type IV Collagenase for 1 hour to yield single cell suspensions. Samples were incubated with anti-CD16/32 (2.4G2, Catalog no. 553141, BD Pharmingen™, San Diego, CA), for 5 minutes at room temperature and subsequently stained with anti-CD45 (clone 30-F11, Catalog no. 103138, BioLegend), anti-CD11b (clone M1/70, Catalog no. 25-0112-82, ThermoFisher Scientific, Waltham, MA), anti-APC (clone-AL21, Catalog no. 560595 BD Pharmingen™), anti-CD64 (Clone X54-5/7.1.1, Catalog no. 558455, BD Pharmingen™), anti-MHC II (Clone M5/114.15.2, Catalog no. 562363, BD Pharmingen™), anti-CD11c (Clone HL3, Catalog no. 553801, BD Pharmingen™), anti-NK1.1 (Clone PK136, Catalog no. 562921, BD Pharmingen™), anti-CD45R/B220 (Clone RA3-6B2, Catalog no. 562922, BD Pharmingen™), anti-CD3e (Clone 145-2c11, Catalog no. 562600, BD Pharmingen™), anti-Ly6G (Clone 1A8, Catalog no. 562737, BD Pharmingen™), or anti-MerTK (Clone 108928, Catalog no. 747837, BD Pharmingen™). Cells were stained with an infra-red fluorescent viability dye (Life Technologies, Grand Island, NY). The gating strategy was as follows: lymphocytes were identified by forward -scatter area (FSC-A) and side scatter area (SSC-A) gates, followed by two singlets gates (FSC-A *vs.* FSC-W and SSC-A *vs.* SSC-W) followed by live/dead identification using the infra-red fluorescent viability dye. Alive CD45^+^ cells were plotted for CD11b^+^ *vs* dump channel (anti-NK1.1/Ly6G/CD3e/CD45R/B220) to remove NK cell, granulocyte, T cell and B cells lineages, respectively, and the CD11b^+^ cells were further gated accordingly. Negative controls consisted of fluorescence minus one splenocytes. Cells were evaluated with either BD LSR II or BD Canto II Benchtop cytometers with BD Diva software version 6.7 (BD Biosciences, San Diego, CA). Final data was analyzed using FlowJo software version 10 (Tree Star Inc., Ashland, OR).

### Cell Sorting and RNA Extraction

Conjunctival epithelium was excised from B6 mice housed under normal non-stressed environmental conditions (NS) or exposed to DS for 5 days (DS5) and different subgroups of CD11b^+^ cells I) CD11b^+^MHCII^+^ II) CD11b^+^MHCII^-^CD64^+^Ly6c^high^ III) CD11b^+^MHCII^-^CD64^+^Ly6c^inter^ IV) CD11b^+^MHCII^-^CD64^+^Ly6c^low/negative^ were sorted using the Aria-II cell sorter at the Baylor College of Medicine cytometry and cell sorting core. Total RNA was extracted using a QIAGEN RNeasy Plus Micro RNA isolation kit (Qiagen) according to manufacturer’s instruction. The concentration and purity of RNA was assessed using a NanoDrop 1000 (ThermoFisher Scientific, Waltham, MA).

### NanoString nCounter Gene Expression Analysis

This was performed by the Genomic and RNA Profiling Core at Baylor College of Medicine. RNA quality was checked using the NanoDrop spectrophotometer and Agilent Bioanalyzer. Using the NanoString nCounter Gene Expression system, total RNA was hybridized with the NanoString Technologies nCounter Gene Expression Mouse Myeloid Innate Immunity V2 Panel codeset (NS_MM_Myeloid_V2.0) containing 770 unique pairs of 35-50bp reporter probes and biotin-labeled capture probes, including internal reference controls (NanoString, Seattle, WA). Overnight hybridization occurred for 20 hours at 65°C. Removal of excess probes with magnetic bead purification was performed on the nCounter Prep Station (software v4.0.11.2) on the High Sensitivity assay. Briefly, the probe-mRNA structure was affinity purified by its 3’ end to remove excess reporter probes, then by its 5’ end to remove excess capture probes. Once unbound probes were washed away, the tripartite structure was bound to the streptavidin-coated cartridge by the biotin capture probe, aligned by an electric current (negative to positive), and immobilized. Photobleaching and fluorophore degradation was prevented with the addition of SlowFade. The cartridge containing immobilized samples was transferred to the nCounter Digital Analyzer (software v3.0.1.4) and scanned at 555 field of view (FOV). An epi-fluorescent microscope and CCD camera identified sets of fluorescent spots, which were tabulated for data output. Quality control metrics were recorded using the nSolver Analysis Software v4.0.6.2. Data from each group was analyzed using nSolver software. Heat maps of the normalized data from all non-stressed cell populations and the monocyte and MHCII+ populations from NS and DS were created with GraphPad Prism V9.0.2 (San Diego, CA).

## Results

### Conjunctival Monocyte Recruitment Is CCR2 Dependent

**Figure 1:**
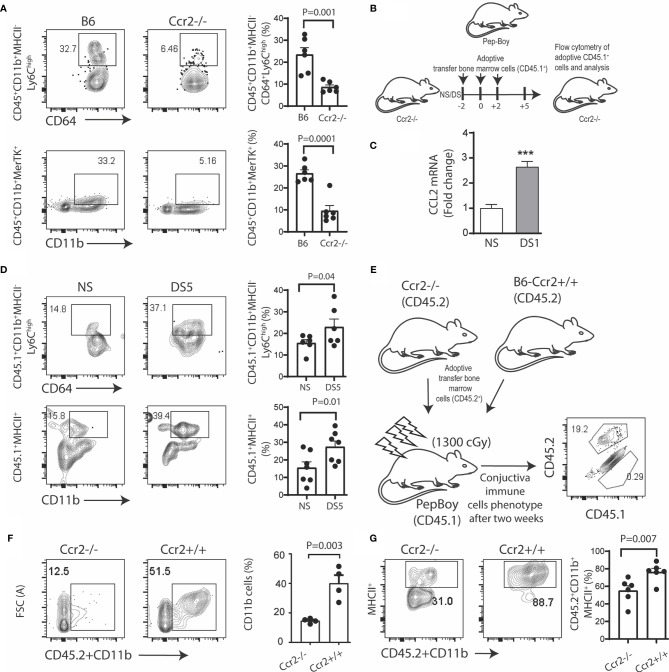
Desiccation stress recruits monocytes into the conjunctiva in a CCR2 dependent manner. The gating strategy used for these experiments is provided in **Supplementary Figure 1A**. **(A)** Single cell suspensions prepared from conjunctivae of C57BL/6 (B6) and Ccr2^-/-^ strains were stained with anti-CD45, CD11b, MHCII, CD64, Ly6c and MerTK. Squares within the representative plots show frequency of CD45^+^CD11b^+^MHCII^-^CD64^+^Ly6C^high^ (classical monocytes) and CD45^+^CD11b^+^MerTK^+^ (tissue resident macrophages) cells (n = 6/group); **(B)** Experimental design, bone marrow cells from Pepc/BoyJ (2x10^6) were adoptively transfer *via* intra-orbital injection at 2 day time intervals and the recipient mice either remained in normal environmental conditions (NS) or were exposed to desiccating stress for 5 days (DS5) (n = 6/group); **(C)** relative fold change in CCL2 mRNA after desiccating stress for 1 day (DS1) (n = 6/group); **(D)** Adoptively transferred cells were tracked by gating on CD45.1^+^, squares within the representative plots showing frequency of classical monocytes (top) and CD45^+^CD11b^+^MHCII^+^ cells (bottom). The bar graphs on the right side indicate the percentages of these cells with each dot representing one animal (n = 6/group); **(E)** Ccr2^-/-^/Ccr2^+/+^ bone marrow chimera. Bone marrow ablation in Pepc/BoyJ mice was accomplished with ^137^Cs irradiation with 1300 cGy, followed by intraorbitally injection of 2x10^6 bone marrow cells from Ccr2^-/-^/CD45.2 (2x10^6) or wild type B6 (Ccr2^+/+^/CD45.2) donors. Flow cytometry scatter plot shows the number of endogenous (CD45.1) or transplanted (CD45.2) immune cells in the conjunctiva two weeks post transfer; **(F)** Donor CD45.2^+^CD11b^+^ myeloid cells were measured in the conjunctiva of Pepc/BoyJ recipient mice after exposure to desiccating stress for 5 days (n = 6/group). **(G)** Donor CD45.2^+^CD11b^+^MHCII^+^ cells were measured in the conjunctiva of Pepc/BoyJ recipient mice after exposure to desiccating stress for 5 days (n = 6/group). The error bars indicate the standard error of mean (SEM), Student T-test was used for statistical comparison. ***p < 0.0001.

To test the hypothesis that myeloid cell recruitment to the conjunctiva is CCR2 dependent, we compared the percentages of monocytes and tissue macrophages in the conjunctiva of CCR2^-/-^ and WT C57BL/6 strains maintained in normal environmental conditions (relative humidity 50-60%). The gating strategy used for these experiments is provided in **Supplementary Figure 1A**. We found the percentages of both classical monocytes (Ly6C^hi^MHCII^-^) and CD11b^+^MERTK^+^ macrophages were significantly reduced in the CCR2^-/-^ conjunctiva compared to WT (**Figure 1A**). We then performed adoptive transfer of CD45.1^+^ bone marrow cells to CD45.2^+^CCR2^-/-^ recipients kept under normal non-stressed environmental conditions (NS) or DS for 5 days (**Figure 1B**). Expression of the CCR2 ligand, CCL2 in the conjunctiva was found to significantly increase after 1 day of DS (**Figure 1C**). CCR2^-/-^ was chosen as the adoptive transfer recipient, because this strain has a reduced number of circulating monocytes and adoptively transferred cells from CCR2^+/+^ donors have been found to have a competitive advantage in recruitment to inflamed tissues (7). We found a significant increase in the percentage of both CD45.1^+^ classical monocytes and MHCII^+^CD11b^+^ cells in the DS-exposed conjunctiva compared to the non-stressed group (**Figure 1D**). Finally, we compared DS-induced myeloid recruitment in chimeric mice in which CD45.1^+^ recipients were reconstituted with bone marrow from congenic CCR2^-/-^ or CCR2^+/+^ strains (**Figure 1E**). There was virtually complete loss of the resident CD45.1^+^ cells in the recipient mice, so all of the cells detected in the conjunctiva were donor derived (**Figure 1E**). Following DS for 5 days, recruitment of total myeloid cells and MHCII^+^ cells to the conjunctiva was significantly greater in the CCR2^+/+^ chimeras (**Figures 1F, G**). These findings indicate the proinflammatory stress of desiccation increases CCL2 expression and recruits myeloid cells to the conjunctiva in a CCR2 dependent fashion.

### DS Triggers a Monocyte to Macrophage Cascade in the Conjunctiva

The fate of the myeloid phagocytic cells in the conjunctiva that participate in DS-induced inflammation has not been investigated. We hypothesized that DS alters conjunctival myeloid cell differentiation and gene expression. To test this, we compared monocyte and macrophage populations in mice maintained under normal humidity with those exposed to DS for 3 and 5 days.

Flow cytometry was used to evaluate changes in CD11b^+^ myeloid cells, MHCII^+^ cells, macrophages (MHCII^+^CD64^+^), monocytes (Ly6C^+^CD64^lo^), and CD11c^+^ cell populations (after excluding T cell, B cell, NK cell and granulocyte lineages) in the conjunctiva in non-stressed conditions (NS) and after DS for 3 and 5 days (DS3 and (DS5). The gating strategy shown in **Supplementary Figure 1A** was used for this experiment. We evaluated the relative percentages of immune cell subsets in the conjunctiva of C57BL/6 mice maintained in a normal environment. CD11b^+^ cells were found to be the predominant cell population. Among these, the majority were myeloid cells, but CD11b was detected on several other cell lineages, including certain T cells, B cells, NK cells and granulocytes (**Supplementary Figure 1B**). These non myeloid lineages were excluded in the dump channel in our gating strategy (**Supplementary Figure 1A**).

**Figure 2:**
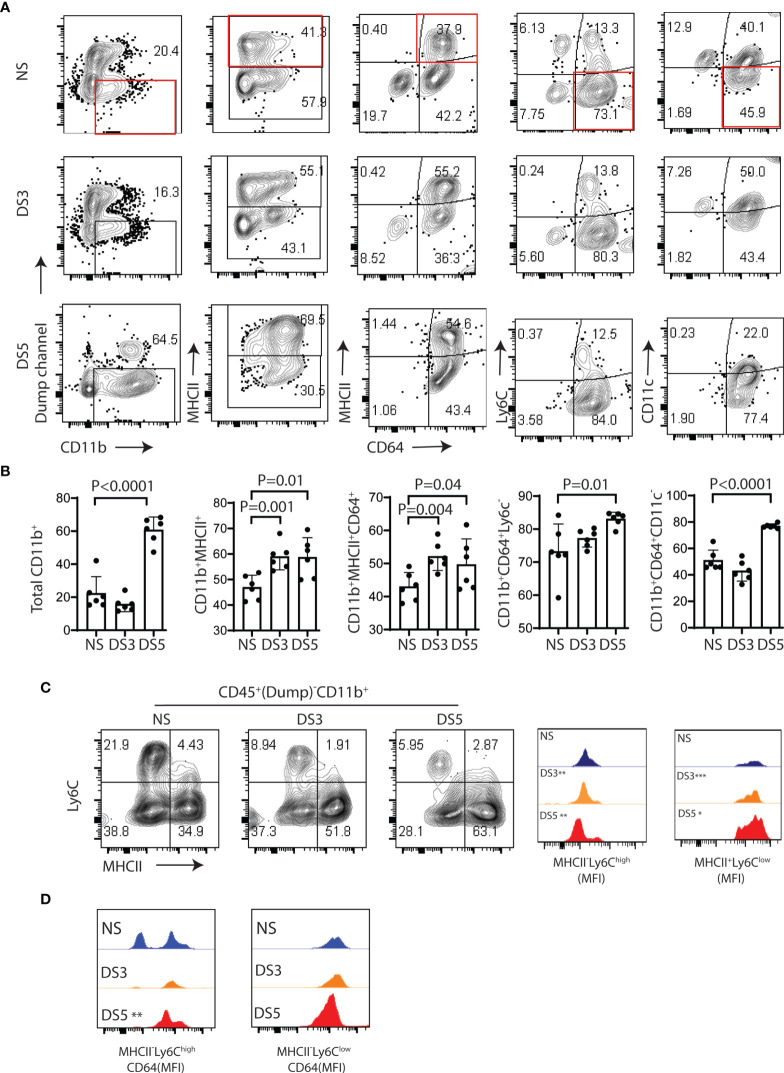
Immune cells phenotype in the conjunctiva of B6 mice following desiccation stress. The gating strategy used for these experiments is provided in **Supplementary Figure 1A**. **(A)** Single cell suspensions prepared from conjunctivae of C57BL/6 (B6) mice housed in normal humidity (NS) or exposed to desiccating stress for 3 (DS3) or 5 (DS5) days were stained with anti-CD45, CD11b, MHCII, CD64, Ly6c and CD11c. A dump channel (anti-NK1.1/Ly6G/CD3e/CD45R/B220) was included to remove NK cell, granulocyte, T cell and B cells lineages. The four quadrants of the representative plots show percentages of the selected population. **(B)** Flow cytometry data from each group are visually presented in the bar graphs (n=6, dots are individual samples). The non-stressed (NS) group was statistically compared with the DS3 or DS5 groups using the Student T-test; **(C)** Representative flow plots of Ly6C and MHCII in the NS, DS3 and DS5 groups (left) with histograms of mean fluorescence intensities (MFI) (right); **(D)** MFI of CD64^+^ cells among MHCII^-^Ly6c^high^ and MHCII^+^Ly6c^low^ population. *p < 0.01, **p < 0.001, ***p < 0.0001.

Significant changes in certain myeloid populations were observed in the groups exposed to DS. By DS5, there was a 40% increase in the percentage of CD11b^+^ myeloid cells, a 10% increase in MHCII^+^ cells and an 8% increase in MHCII^+^CD64^+^ macrophages. In contrast, there were significant decreases in Ly6C^+^CD64^-^ monocytes, CD11c^+^CD64^lo^ and CD11c^+^MHCII^+^cells, respectively (**Figures 2A, B** and **Supplementary Figures 2A, B**). The ratio of myeloid CD11b^+^ relative to the lineages in the dump channel also increased (**Supplementary Figure 2C**). There was a progressive decrease in mean fluorescent intensity of Ly6C^hi^MHC^lo^ (immature monocytes) and progressive increase in Ly6C^lo^MHCII^hi^ (putative antigen presenting cells) from NS to DS5 (**Figure 2C**). The majority of Ly6C^hi^MHC^lo^ cells were CD64^+^ with 2 peaks noted in NS and an increased percentage with one peak in the DS group (**Figure 2D**).

These findings indicate that DS promotes a monocyte to macrophage cascade and increases the percentage of MHCII^+^ myeloid cells that may be capable of antigen presentation in the conjunctiva.

### DS Alters Gene Expression in Myeloid Cell Populations in the Conjunctiva

**Figure 3:**
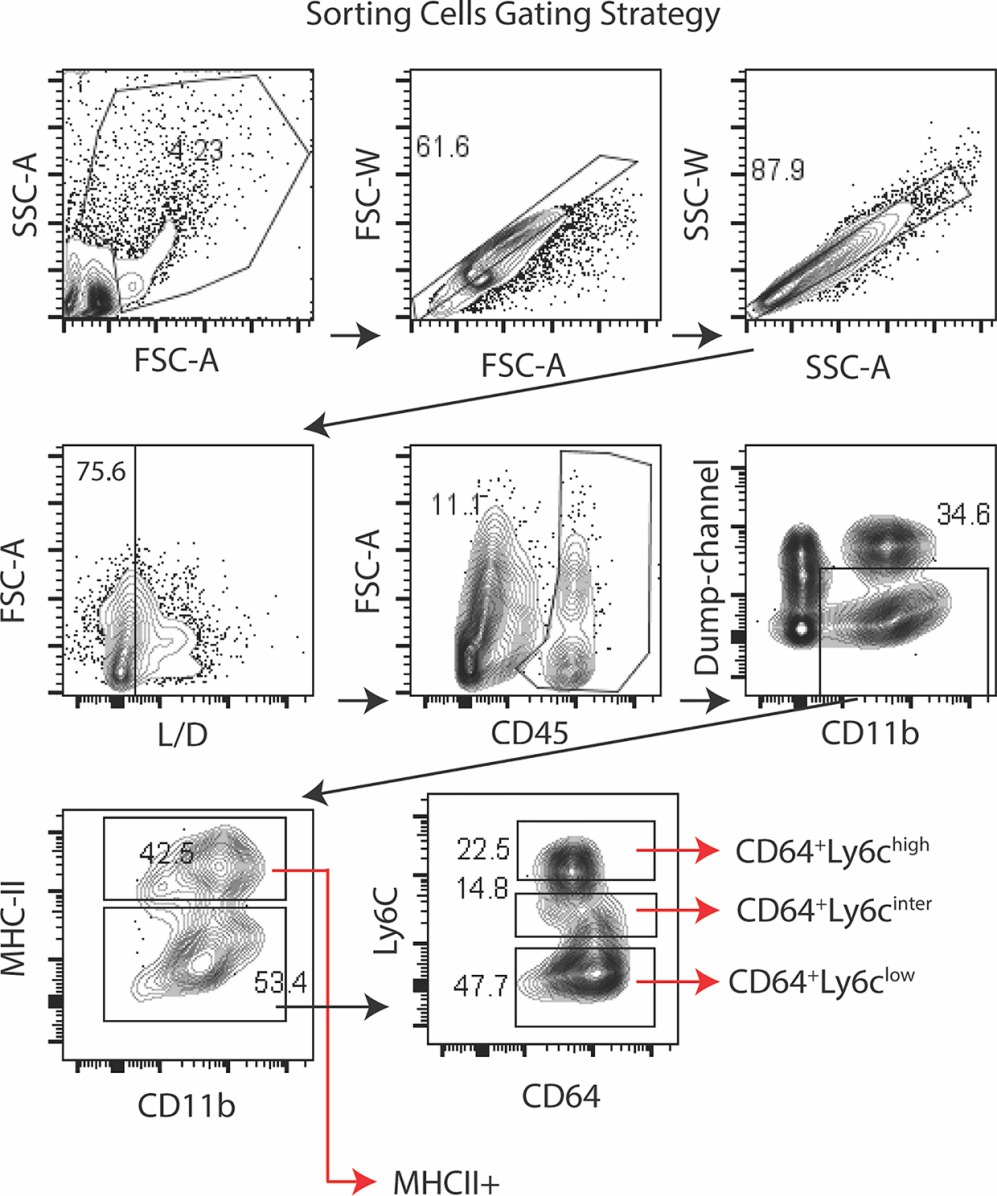
Sorting strategy for Nanostring arrays. Lymphocytes were identified by forward -scatter area (FSC-A) and side scatter area (SSC-A) gates, followed by two singlets gates (FSC-A *vs.* FSC-W and SSC-A *vs.* SSC-W) followed by live/dead identification using the infra-red fluorescent viability dye. Alive CD45^+^ cells were plotted for CD11b^+^ *vs* dump channel (anti-NK1.1/Ly6G/CD3e/CD45R/B220) to remove NK cell, granulocyte, T cell and B cells lineages, respectively. The CD11b^+^ cells were further gated on MHCII. The MHCII^-^ population was sub-gated on Ly6C as MHCII^-^CD64^+^Ly6c^high^ (monocytes), MHCII^-^CD64^+^Ly6c^inter^ and MHCII^-^CD64^+^Ly6c^low^ (macrophages). Cells were sorted from twenty C57BL/6 mice (n = 20) to obtain sufficient RNA to perform the NanoString expression profiles.

**Figure 4:**
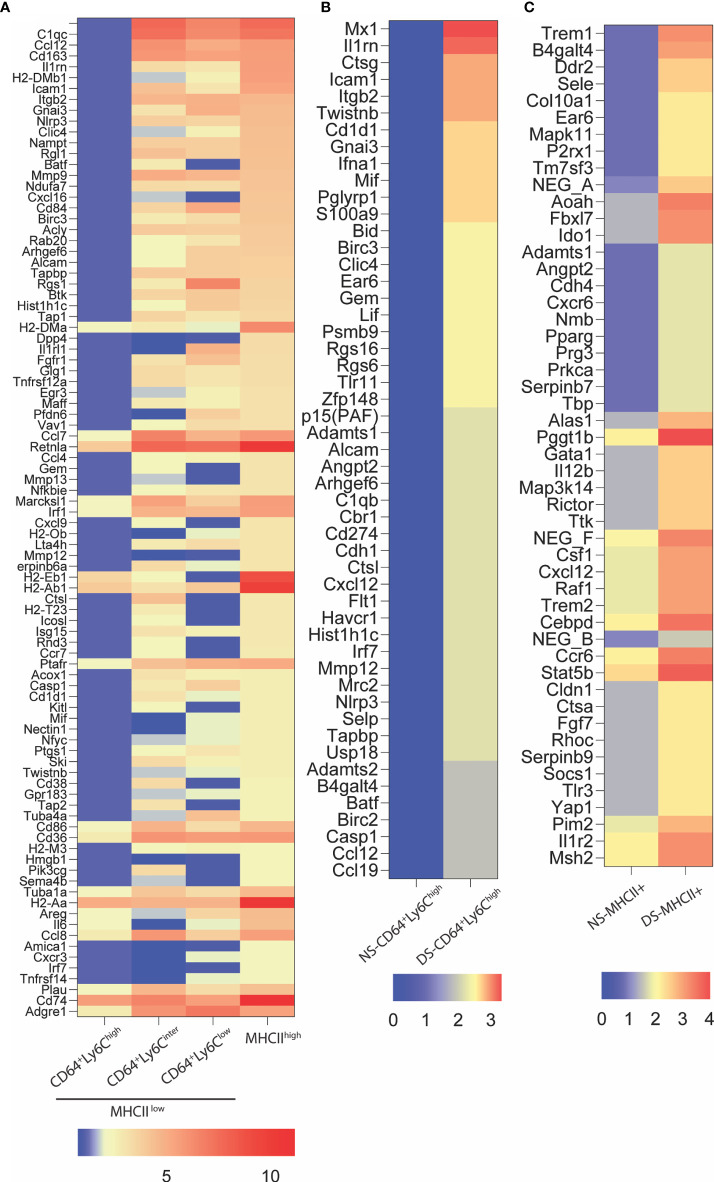
NanoString nCounter gene expression analysis. **(A)** Heatmap representation of top 94 genes upregulated ≥ 2 log^2^ fold in MHCII^+^ group as compared to MHCII^-^CD64^+^Ly6c^high^ (monocytes). These genes are involved in antigen presentation, lymphocytes trafficking, cytokine and chemokine signaling and myeloid cell differentiation; **(B)** Relative gene expression of top fifty upregulated genes in MHCII^-^CD64^+^Ly6c^high^ (monocyte) cells exposed to 5 days of dessicating stress (DS5) as compared to control (NS); **(C)** Relative gene expression of top 50 genes upregulated in MHCII^+^ cells exposed to dessication stress (DS5) as compared to control. The NanoString data was analyzed using the nSolver Analysis Software v4.0.6.2 and the heat maps were generated using GraphPad Prism V9.0.2 (San Diego, CA).

Gene expression in the conjunctiva was profiled using the NanoString nCounter mouse myeloid innate immunity V2 panel in 4 populations of cells sorted by the strategy shown in **Figure 3**. These include one MHCII^hi^, and three MHCII^lo^ populations consisting of Ly6C^hi^, Ly6C^inter^, and Ly6C^lo^ cells taken from non-stressed (NS) and DS5 groups. Cells sorted from both conjunctivae of 20 mice were required to obtain sufficient RNA to perform one expression panel. Data from these pooled samples are shown in **Figure 4**. Comparison of gene expression between the MHCII^-^ monocyte and MHCII^+^ populations in non-stressed conjunctiva revealed a ≥ 2 log^2^ fold increase in 95 genes (**Figure 4A**) and decrease in 46 genes. Upregulated genes were associated with antigen presentation (MHCII, CD74, CD86), cytokine/chemokine signaling, myeloid cell differentiation and innate inflammation. Expression was also compared between NS and DS monocyte and MHCII^+^ populations (**Figures 4B, C**). DS increased innate inflammatory genes in both monocytes and MHCII^+^ cells and increased M1 macrophage (Trem1, Ido1, Il12b, Stat5b) and decreased homeostatic (Mertk) and M2 macrophage (Arg1, CCL24, **Supplementary Figure 2D**) genes in the MHCII^+^ cells. These findings suggest there are myeloid cell populations in the conjunctiva with distinct phenotype and gene expression patterns. DS recruits myeloid cells from the blood to the conjunctiva and significantly changes their phenotype and gene expression profile.

## Discussion

This study evaluated myeloid cells populations in the conjunctiva in normal non-stressed environmental conditions and in the desiccating stress dry eye model that has features similar to SS KCS (marked reduction in tear volume and secretion, loss of reflex tearing, loss of goblet cells). In non-stressed homeostatic conditions, we found distinct myeloid populations ranging from classical Ly6C^hi^ monocytes to macrophages. Our findings indicate that recruitment of myeloid cells to the conjunctiva in homeostasis and during DS is CCR2 dependent. This may be in response to CCL2 which was found to increase in response to DS or other CCR2 ligands (CCL7, 8, 12 and 13) (21). DS changed the ratio of myeloid cell populations, causing a decrease in classical monocytes and increase in MHCII^+^CD64^+^ macrophages. In this DS induced monocyte cascade, there was a concurrent increase in MHCII^+^ and CD64^+^ cells as Ly6C^+^ monocytes decreased. These findings suggest that the inflammatory milieu created by DS promotes maturation of monocytes to macrophages, potentially with antigen presenting capability. We did not determine if in addition to recruitment, these changes in myeloid cell populations could be due to proliferation or increased death of these cells *in situ*. We have previously reported that DS promotes apoptosis of the ocular surface epithelia (22). The findings in our mouse model are consistent with the increased percentage of HLA-DR^+^ cells found in the conjunctival epithelium of patients with non-SS and SS KCS (11, 23–25). HLA-DR expression was found to positively correlate with categorical severity of KCS and inversely correlate with conjunctival goblet cell density in SS in one study (11).

Mature APCs have been found to be essential in the induction of autoreactive T cells in the mouse DS model (14). We previously reported that generation of autoreactive T cells is suppressed by clodronate depletion of phagocytes on the ocular surface (13). The findings of our current study suggest these APCs may be induced from resident and recruited monocytes when the ocular surface is under desiccating stress.

Changes in gene expression in the monocyte to macrophage cascade are consistent with monocyte maturation to macrophages with increased levels of MHCII and antigen processing and presentation genes, as well as a variety of innate inflammatory genes (complement factors, NLRP3, caspase 1, matrix metalloproteinases, cytokines and chemokines).

DS-induced changes in gene expression profiles were seen in tandem to the changes in myeloid cell populations observed by flow cytometry. In the monocyte population, there was increased expression of a number of genes associated with monocyte differentiation and activation. Of note, two genes associated with the NLRP3 inflammasome (Nlrp3 and Casp1) were among those with the greatest increase with DS. There was also increased expression of Cd1d1, the glycoprotein that presents lipids to and activates Type 1 NKT cells (26). Expression of p38 MAPK (Mapk11), MAPK3k14 and M1 macrophage associated genes (Trem1, Ido1, Il12b, Stat5b) increased in DS stimulated MHCII^+^ cells. IFN*γ* has previously been implicated in promoting goblet cell loss in KCS (16, 19, 27) and increased expression in the conjunctiva and concentration in tears has been found in SS (19, 28–32) Induction of IFN*γ* in NK and T cells requires IL-12 and IL-18. These findings suggest that increased expression of these IFN*γ* inducing cytokines in DS activated myeloid cells could contribute to IFN*γ* mediated goblet cell loss.

The conjunctiva presents a unique challenge to characterize immune cells because only a small number of myeloid cells can be sorted from the mouse conjunctiva and a large sample size was required to obtain sufficient RNA to perform the expression panel. Therefore, our results represent RNA extracted from cell populations pooled from both eyes of 20 mice per group. A robust threshold of ≥ 2 log^2^ fold difference was used for comparing differentially expressed genes between monocytes and MHCII^+^ cells in NS and ≥ 1.5 log^2^ fold difference was used for the NS to DS comparisons. The gene expression findings in this study will need to be confirmed using replicates or by single cell sequencing in the future.

Our findings suggest that monocyte recruitment is a consequence of reduced lacrimal function and ocular surface desiccation, as occurs in SS. They also indicate that monocytes subjected to DS become activated and produce dry eye associated inflammatory mediators and MHCII antigens that may promote development or amplify severity of KCS. This concept is consistent with the finding that MHCII is a sensitive biomarker that correlates with severity of human KCS (25). Additionally, we found that five of the differentially expressed genes in DS stimulated monocytes (Mx1, Irf7, Baft, S100a9 and Icam1) are all found to have significantly increased expression in the conjunctiva of SS patients (manuscript submitted to this special issue). Further research is needed to determine if monocytes or macrophages in the conjunctiva express these KCS biomarkers. Minimizing desiccation of the ocular surface in dry eye could potentially reduce myeloid cell recruitment and the downstream events. Approved dry eye therapies target certain inflammatory mediators produced by DS activated myeloid cells. Lifitigrast is an antagonist of the integrin LFA, a ligand of the adhesion molecule ICAM-1, that showed increased expression in the DS treated MHCII^+^ cells (33). A proposed mechanism of action of lifitigrast is suppression of myeloid cell recruitment and migration into the conjunctiva. Corticosteroids inhibit both NFkB signaling and activation of the NLRP3 inflammasome (34), pathways involved in dry eye associated inflammation and that increase in DS activated monocytes (30, 35). Taken together, this data suggests that myeloid cells may be important in the pathogenesis of KCS as well as relevant therapeutic targets.

## Data Availability Statement

The datasets presented in this study can be found in online repositories. The names of the repository/repositories and accession number(s) can be found below: NCBI GEO; GSE176390.

## Ethics Statement

The animal study was reviewed and approved by Institutional Animal Care and Use Committee at Baylor College of Medicine.

## Author Contributions

JA, SP, and CP were involved in conception and design of the study. JA and SP were involved in data acquisition. JA, SP, and CP were involved in data analysis and interpretation. JA and SP drafted the manuscript. All authors contributed to the article and approved the submitted version.

## Funding

This work was supported by NIH Grant EY11915 (SP), NIH Core Grant EY002520, the Cytometry and Cell Sorting Core at Baylor College of Medicine with funding from the CPRIT Core Facility Support Award (CPRIT-RP180672), the NIH grant (CA125123) and the assistance of Joel M. Sederstrom, and the BCM Genomic & RNA Profiling Core (GARP) [P30 Digestive Disease Center Support Grant (NIDDK-DK56338) and P30 Cancer Center Support Grant (NCI-CA125123), NIH S10 grant (1S10OD02346901)]. Additional support includes an unrestricted grant from Research to Prevent Blindness, New York, NY (SP), The Hamill Foundation, Houston, TX (SP), The Knights Templar Eye Foundation, Flower Mound, TX and the Sid W. Richardson Foundation, Ft Worth, TX (SP).

## Conflict of Interest

The authors declare that the research was conducted in the absence of any commercial or financial relationships that could be construed as a potential conflict of interest.

## Supplementary Material

Supplementary Figure 1
**(A)** Flow cytometry sorting strategy used for experiments show in **Figures 1** and **2**. Lymphocytes were identified by forward -scatter area (FSC-A) and side scatter area (SSC-A) gates, followed by two singlets gates (FSC-A *vs.* FSC-H and SSC-A *vs.* SSC-H) followed by live/dead identification using the infra-red fluorescent viability dye. Live CD45^+^ cells were plotted for CD11b^+^ *vs* dump channel (anti-NK1.1/Ly6G/CD3e/CD45R/B220) to remove NK cell, granulocyte, T cell and B cells lineages, respectively. The CD11b+ cells were further gated on MHCII, CD64, Ly6C and CD11c. **(B)** Immune cell populations in the normal C57BL/6 conjunctiva expressed as a percentage of total CD45^+^ cells. CD11b^+^ non-myeloid cell lineages are shown on the right side.

Supplementary Figure 2
**(A)** Flow cytometry data shown in **Figure 2A**. from each group are visually presented as concentric donuts (n = 6). The non-stressed (NS) group was statistically compared with the 3- and 5-day desiccating stress (DS3 or DS5) groups using the Student T-test; **(B)** Representative flow cytometry dot plots (left) and bar graphs showing individual samples (n=6, right) of MHCII+CD11c^+^ cells. The non-stressed (NS) group was statistically compared with the 3- and 5-day desiccating stress (DS3 or DS5) groups using the Student T-test; **(C)** Bar graph shows ratio of CD11b^+^ myeloid cells (mCD11b^+^) to non-myeloid lineage CD11b^+^ cells in the dump channel. Dots are individual samples; **(D)** Heatmaps showing expression levels of M2 macrophage markers in CD64^+^Ly6chigh and MHCII^+^ populations in non-stressed (NS) conditions and after DS5.
