# Supplementary figures and images for: Desiccation Induced Conjunctival Monocyte Recruitment and Activation - Implications for Keratoconjunctivitis
Source: Front Immunol. 2021 Jul 8;12:701415. doi: 10.3389/fimmu.2021.701415 (PMC8297564; doi:10.3389/fimmu.2021.701415)

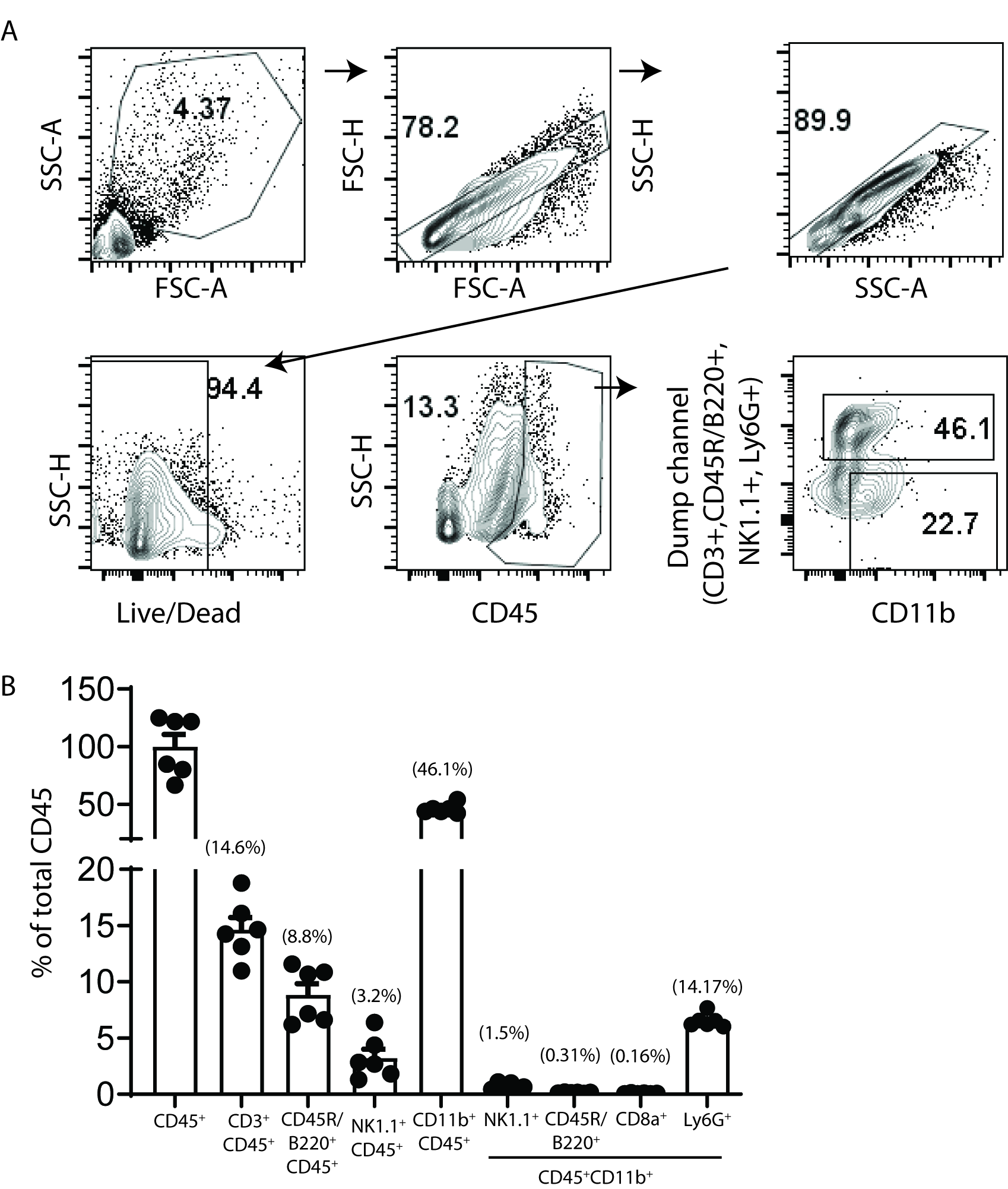

Supplement: Supplementary Figure 1 — (A) Flow cytometry sorting strategy used for experiments show in Figures 1 and 2 . Lymphocytes were identified by forward -scatter area (FSC-A) and side scatter area (SSC-A) gates, followed by two singlets gates (FSC-A vs. FSC-H and SSC-A vs. SSC-H) followed by live/dead identification using the infra-red fluorescent viability dye. Live CD45+ cells were plotted for CD11b+ vs dump channel (anti-NK1.1/Ly6G/CD3e/CD45R/B220) to remove NK cell, granulocyte, T cell and B cells lineages, respectively. The CD11b+ cells were further gated on MHCII, CD64, Ly6C and CD11c. (B) Immune cell populations in the normal C57BL/6 conjunctiva expressed as a percentage of total CD45+ cells. CD11b+ non-myeloid cell lineages are shown on the right side. [file Image_1.tif]

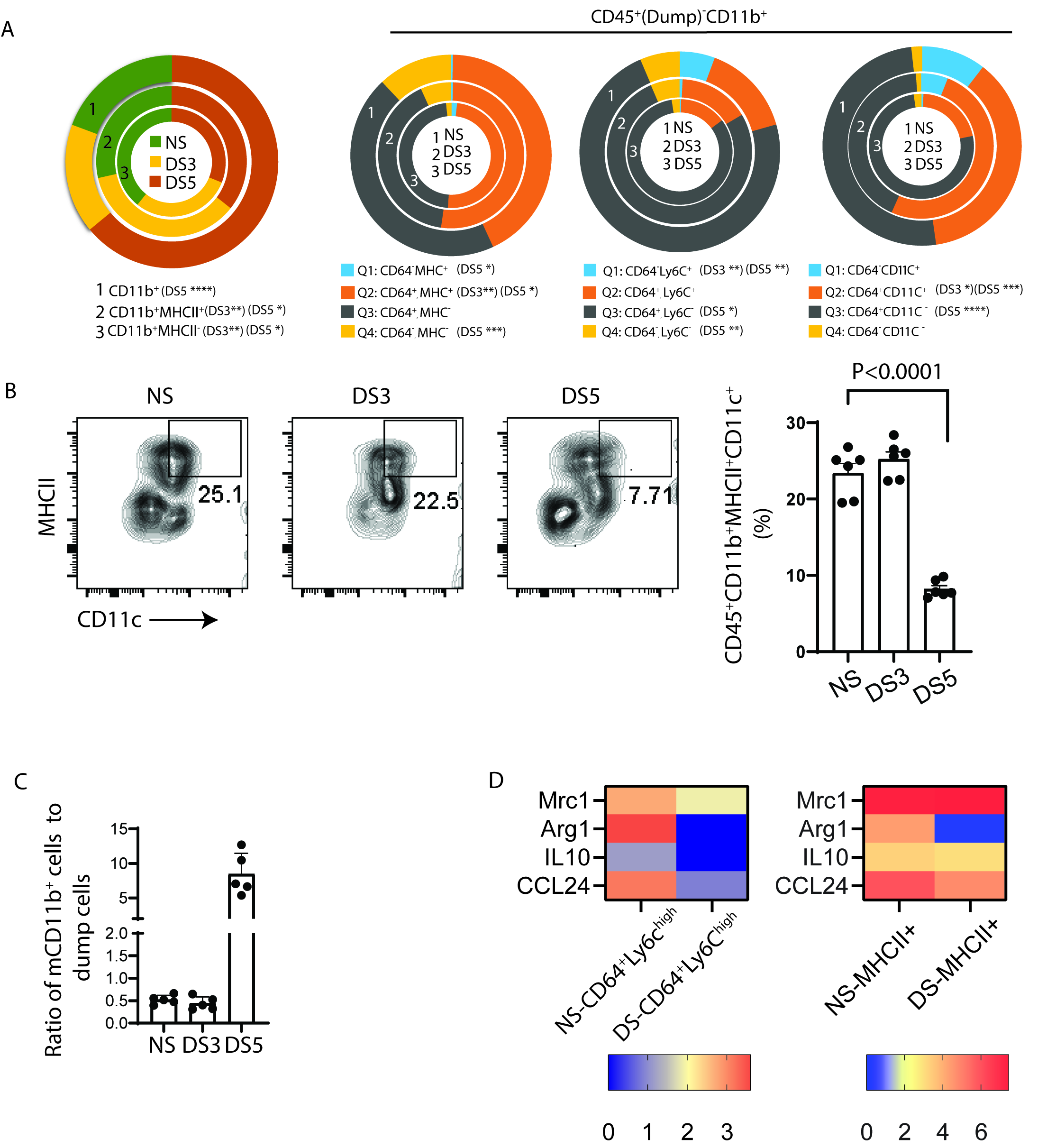

Supplement: Supplementary Figure 2 — (A) Flow cytometry data shown in Figure 2A . from each group are visually presented as concentric donuts (n = 6). The non-stressed (NS) group was statistically compared with the 3- and 5-day desiccating stress (DS3 or DS5) groups using the Student T-test; (B) Representative flow cytometry dot plots (left) and bar graphs showing individual samples (n=6, right) of MHCII+CD11c+ cells. The non-stressed (NS) group was statistically compared with the 3- and 5-day desiccating stress (DS3 or DS5) groups using the Student T-test; (C) Bar graph shows ratio of CD11b+ myeloid cells (mCD11b+) to non-myeloid lineage CD11b+ cells in the dump channel. Dots are individual samples; (D) Heatmaps showing expression levels of M2 macrophage markers in CD64+Ly6chigh and MHCII+ populations in non-stressed (NS) conditions and after DS5. [file Image_2.tif]
